# Craniofacial Osteomas: From Diagnosis to Therapy

**DOI:** 10.3390/jcm10235584

**Published:** 2021-11-27

**Authors:** Achille Tarsitano, Francesco Ricotta, Paolo Spinnato, Anna Maria Chiesa, Maddalena Di Carlo, Anna Parmeggiani, Marco Miceli, Giancarlo Facchini

**Affiliations:** 1 Maxillofacial Surgery Unit, IRCCS Policlinico di S. Orsola, 40138 Bologna, Italy; achille.tarsitano2@unibo.it (A.T.); francesco.ricotta@aosp.bo.it (F.R.); 2 Diagnostic and Interventional Radiology Unit, IRCCS Istituto Ortopedico Rizzoli, 40136 Bologna, Italy; paolo.spinnato@ior.it (P.S.); annamaria.chiesa@ior.it (A.M.C.); maddalena.dicarlo@ior.it (M.D.C.); marco.miceli@ior.it (M.M.); giancarlo.facchini@ior.it (G.F.)

**Keywords:** osteoma, bone neoplasms, oral surgery, computed tomography, radiography

## Abstract

An osteoma is a benign bone lesion with no clear pathogenesis, almost exclusive to the craniofacial area. Osteomas show very slow continuous growth, even in adulthood, unlike other bony lesions. Since these lesions are frequently asymptomatic, the diagnosis is usually made by plain radiography or by a computed tomography (CT) scan performed for other reasons. Rarely, the extensive growth could determine aesthetic or functional problems that vary according to different locations. Radiographically, osteomas appear as radiopaque lesions similar to bone cortex, and may determine bone expansion. Cone beam CT is the optimal imaging modality for assessing the relationship between osteomas and adjacent structures, and for surgical planning. The differential diagnosis includes several inflammatory and tumoral pathologies, but the typical craniofacial location may aid in the diagnosis. Due to the benign nature of osteomas, surgical treatment is limited to symptomatic lesions. Radical surgical resection is the gold standard therapy; it is based on a minimally invasive surgical approach with the aim of achieving an optimal cosmetic result. Reconstructive surgery for an osteoma is quite infrequent and reserved for patients with large central osteomas, such as big mandibular or maxillary lesions. In this regard, computer-assisted surgery guarantees better outcomes, providing the possibility of preoperative simulation of demolitive and reconstructive surgery.

## 1. Introduction and Pathogenesis

An osteoma is a benign lesion characterized by the presence of both cortical and cancellous bone tissue. Its onset is almost exclusively in the craniofacial area [1,2,3,4,5,6].

**Figure 1:**
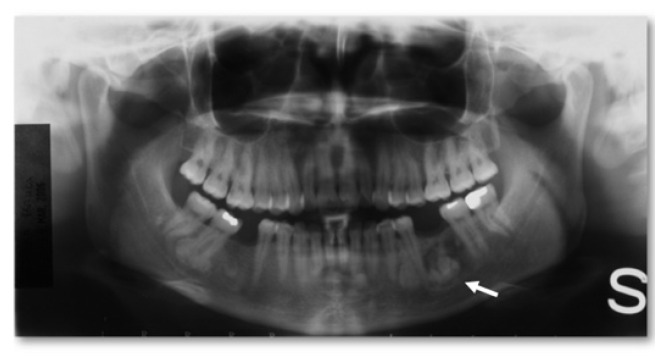
Central osteoma: panoramic radiograph showing a localized, well-defined radiopacity involving the alveolar bone of the left mandibular body (white arrow). The finding was incidental, and the patient did not refer to any symptoms.

**Figure 2:**
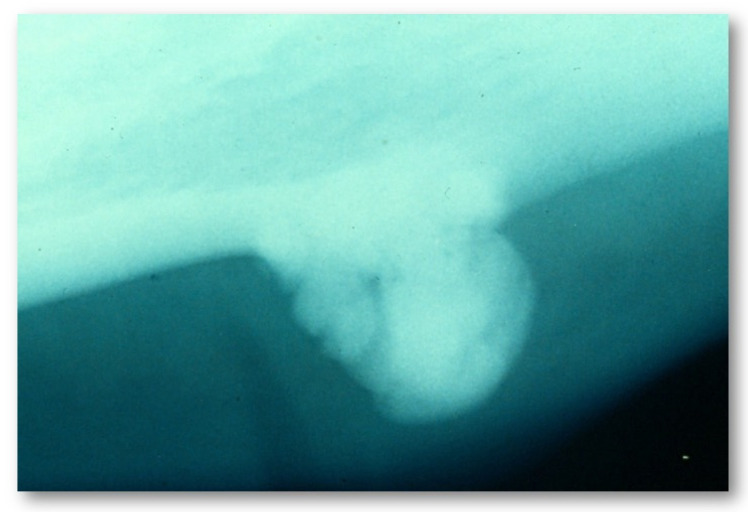
Plain radiography showing a peripheral osteoma involving the mandibular body.

Depending on the site, three subtypes of osteomas are distinguished:

- Central, characterized by progressive endosseous development, eventually resulting in the complete replacement of the affected bone segment (Figure 1);
- Peripheral, consisting of periosteal development that can appear as a pedunculated mass (Figure 2);
- Extraosseous, which develops within the soft tissues, particularly in the muscles [2].

The pathogenesis of osteomas is still under debate, and different onset sites are described in the literature, such as the frontoethmoidal junction or the temporal bone, where osteomas can be associated with congenital cholesteatoma [7,8]. For this reason, some authors consider osteomas to be congenital lesions, arising from an embryonal cartilaginous rest or a persistent embryological periosteum [9]. The association of osteomas with colonic diseases such as Gardner’s syndrome suggests a possible hereditary nature [10]. On the other hand, some of the most frequent osteoma onset locations are susceptible to trauma (e.g., to the frontal bone or to the angle and lower border of the mandible), suggesting that previous trauma may contribute to the development of these tumors [11].

Osteomas exhibit continuous growth rather than growth cessation. This characteristic is the major feature distinguishing them from other bony exostoses such as tori, which are non-pathological protuberances arising from cortical bone with a wide attachment base, usually found in the oral region or, less frequently, in the auditory canal or maxilla [12]. Oral tori are classified according to the onset site, as in torus palatinus and torus mandibularis; the first occurs along the midline of the palate, while the second is often bilateral and is located on the lingual side of the mandible, in the canine or premolar region above the attachment of the mylohyoid muscle [12]. Moreover, while osteomas show a focal implant on the bone, tori have diffuse attachments. Osteomas’ slow growth rate can become faster in cases of osteogenesis rate increases [4]. As they are frequently small and asymptomatic, it is difficult to precisely define osteomas’ exact incidence, which is estimated to be from 0.002 to 3%, with a predilection for occurring in young males (15–30 years) [13].

## 2. Histological Findings

**Figure 3:**
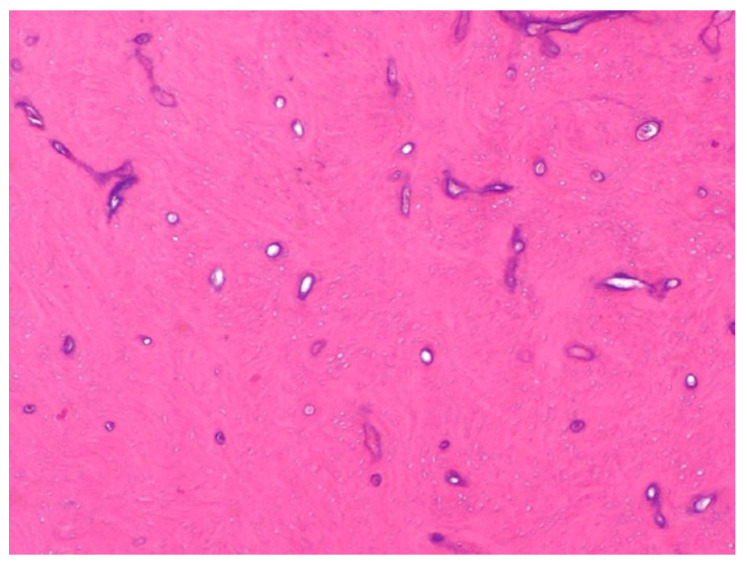
Microscopic view (hematoxylin–eosin staining ×40) of a maxillary sinus osteoma. The following features are observed: multiple areas of compact lamellar bone deposition and proliferation of irregular trabeculae, with few osteons and minimal marrow spaces.

Histologically, an osteoma is represented as a mass of abnormal dense bone that may originate from the periosteum or from bone marrow, thus differentiating between two types of osteomas [1,2]. Compact osteomas, also called “ivory”, are made of mature lamellar bone with minimal marrow spaces and occasional haversian canals without any fibrous structure [3,4] (Figure 3). On the other hand, trabecular osteomas, also called “mature”, are composed of cancellous trabecular bone with bone marrow enclosed by a cortical bone margin [5]. This distinction is typical of all osteomas and does not involve any association with a pattern of tumor proliferation.

The theory of “zonation of histology” is cited in some studies, describing two different areas within osteomas: a fibrous central area, rich in osteoblasts and blood vessels, actively growing from the center to the periphery, and a peripheral area, less vascularized and metabolically active [14]. This difference could justify a possible partial resection only for the proliferative center of the lesion, preventing its growth, notwithstanding that the literature reports some cases of recurrence only after partial treatment [15].

## 3. Clinical Features

**Figure 4:**
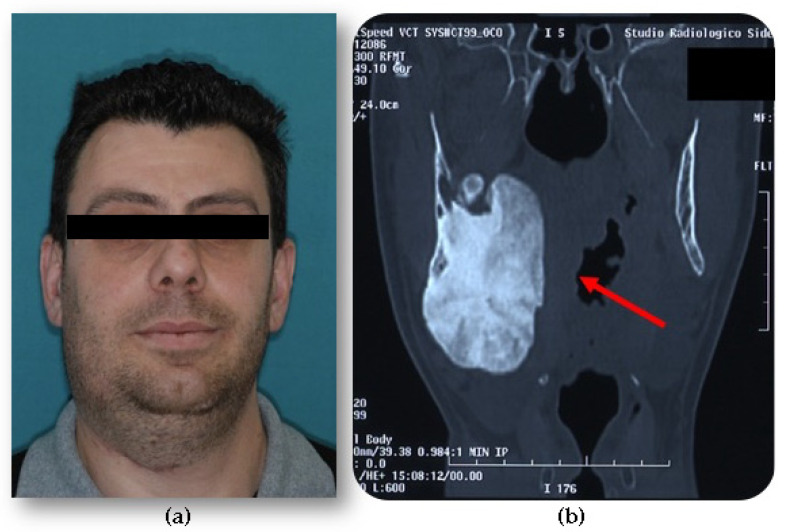
(**a**) A young male affected by an extensive right mandibular osteoma, causing facial swelling. (**b**) Computed tomography (CT) scan shows diffuse enlargement of the entire right mandible, extending to the lateral and medial sides and causing deformation of the pharyngeal walls.

In most cases, osteomas are asymptomatic, and the diagnosis is often made incidentally through radiological investigations conducted for other reasons. More rarely, osteomas can assume significant dimensions causing aesthetic and/or functional problems due to bone distortion, with possible compression of nearby structures (Figure 4). Clinical manifestations of craniofacial osteomas are highly variable according to the sites of onset [4,14], as suggested by multiple case reports described in the literature, the most recent of which we provide for illustrative purposes in Table 1.

**Figure 5:**
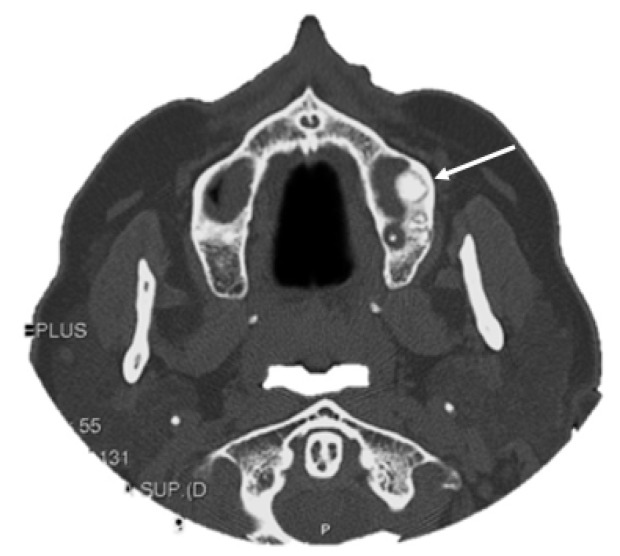
CT scan showing the incidental finding of a small osteoma located in the floor of the left maxillary sinus (white arrow).

The most frequent osteoma onset sites are the jaw and the paranasal sinuses (frontal, ethmoid, maxillary, and sphenoid), followed by the internal and external cranial planking and the maxillary bone [4,15] (Figure 5).

**Figure 6:**
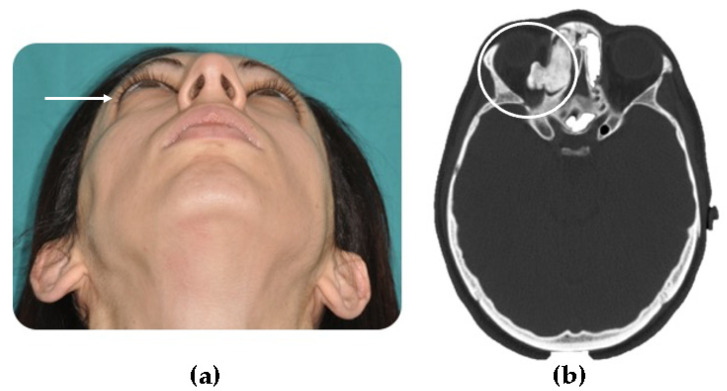
(**a**) A young patient presenting right exophthalmos (white arrow) due to an ethmoid osteoma with orbital invasion; (**b**) CT scan shows an extensive right ethmoid osteoma which invades the orbital cavity (circled in CT scan).

In cases of involvement of the paranasal sinuses, an osteoma occupies the ostiomeatal complex, causing an interruption of mucus drainage and airflow, which clinically manifests with sinusitis, pain, headache, and nasal obstruction [4]. The involvement of the midface with an osteoma can sometimes cause facial asymmetry [16,17,18]; when an osteoma is localized into the orbit, it can cause exophthalmos (Figure 6).

**Table 1:** Literature review of the cases of solitary craniofacial osteomas reported from January 2020 to September 2021, with descriptions of clinical and diagnostic features. (F) female; (M) male; (CT) computed tomography; (CBCT) cone beam CT; (XR) radiography; (MRI) magnetic resonance imaging.

Author	N° Ref.	Patient Gender	Patient Age	Osteoma Localization	Clinic	Imaging	Comorbidity
Ortega Beltrá	[19]	M	68	Mandibula	Ankylosis of the temporomandibular joint	CT	No
Alkhaldi	[20]	M	44	Ethmoid sinus, orbital cavity, ostium of the maxillary sinus	Chronic rhinosinusitis	CT	Prior endoscopic sinus surgery
Dedushi	[21]	M	61	Frontal sinus	Headaches, generalized seizures, transient motor aphasia, regressive hemiparesis, and fluctuating blood pressure values	MRI	No
Ali	[22]	M	35	Frontal sinus	Altered Sensorium	CT, MRI	No
Mlouka	[23]	M	26	Maxillary sinus	Asymptomatic	CBCT	No
Öztürk	[24]	M	15	Frontal sinus	Frontal sinusitis	CT	No
Benzagmout	[25]	M	34	Frontoethmoidal sinus	Swelling, headaches, seizures	CT, MRI	No
Bagheri	[26]	F	30	Frontoethmoidal sinus	Orbital cellulitis	CT	No
Devaraja	[27]	M	21	Frontal sinus	Eyelid swelling and inability to open the eye	CT	No
Nakagawa	[28]	M	27	Frontoethmoidal sinus, anterior cranial fossa and orbit, frontal lobe	Headache and generalized convulsion	CT, MRI	No
Aksakal	[29]	M	53	Frontal sinus	Headache	CT	No
Demircan	[30]	M	17	Mandibular ramus	Swelling, facial asymmetry	XR, CBCT	Prior trauma
Azevedo	[31]	M	30	Nasal fossa, the bilateral ethmoidal cells, and the frontal Sinuses	Swelling	CT, MRI	Prior trauma
Yazici	[32]	F	30	Frontoethmoidal sinus, maxillary sinus, middle concha	Headache, facial pain, and blurring vision	CT	No
Kim	[33]	F	39	Zigomatic bone	Facial swelling	CT	No
Chen	[34]	M	19	Fronto-ethmoid sinus	Diplopia, proptosis	CT	No
Voicu	[35]	M	38	Frontal sinus	Frontal peri-orbital pain	XR, MRI	No
Hania	[36]	M	15	Maxillary sinus	Spontaneous epistaxis	XR, CT	No
Pathak	[37]	M	45	Fronto-ethmoid sinus	Change of behavior, forgetfulness	CT, MRI	No
Lee	[38]	F	23	External auditory canal	Aural fullness	CT	No
Lee	[38]	M	19	External auditory canal	Mild aural fullness	CT	No
Borissova	[39]	F	48	Retromastoid portion of the temporal bone	Facial swelling	CBCT	No
Temirbekov	[40]	F	25	Middle ear, mesotympanum, and hypotympanum	Hearing loss and fullness in the ear	CT	Prior unilateral otitis media
Canzi	[41]	F	64	Eustachian tube of the temporal bone	Progressive bilateral asymmetric hearing loss	CT	No
Falcioni	[42]	F	36	Middle ear, promontory, umbus	Progressive monoliteral hearing loss	CT	No
Lee	[43]	M	24	Ethmoid sinus, medial wall of the orbit	Eye pain, swelling, decreased vision, purulent drainage	CT	No
Saylisoy	[44]	F	53	Eustachian tube of the temporal bone	Intermittent otalgia and otorrhea	CT	No
Tan	[45]	F	40	Temporal bone (retromastoid)	Swelling behind the ear	CT	No
Nilesh	[46]	F	65	Mandibular condyle	Limited mouth opening	XR, CT	No
Ghita	[47]	F	25	Posterior mandible	Facial swelling	XR, CBCT	No
Kayaci	[48]	F	80	Posterolateral wall of the lesser wing of the sphenoid bone	Vision loss, pain, headache	CT	No
Torres	[49]	M	21	Posterior mandible	Facial swelling	CT	No
Nayak	[50]	M	30	Posterior mandible	Swelling in the lower left back tooth region	XR	No
Lazar	[51]	M	33	Posterior mandible	Swelling, airway deviation	CT	No
Guerra	[52]	M	25	Frontal sinus, ethmoid sinus, upper and medial orbital walls	Double vision, progressive change in the positioning of the eye	CT	Prior orbit zygomatic fracture reconstruction due to facial trauma

**Figure 7:**
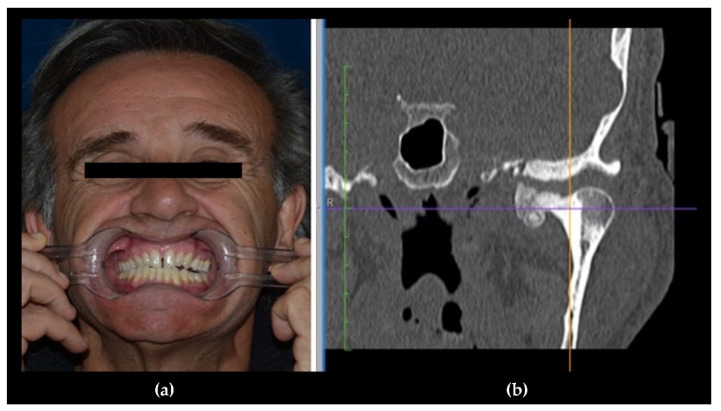
(**a**) A male patient with dental malocclusion, characterized by left open bite and right cross-bite; (**b**) CT scan shows a left condylar osteoma.

In cases of mandibular condyle involvement, the growth of an osteoma can determine a series of dysfunctions. Specifically, it may cause malocclusion, temporomandibular joint (TMJ) functional impairment, limited mouth opening due to ankylosis, and in some rare cases tinnitus and deafness [45,46] (Figure 7).

If multiple facial lesions are present, it is advisable to perform a total body scan by computed tomography (CT), and a colonoscopy to exclude Gardner’s syndrome, which is an autosomal dominant autoimmune disease characterized by intestinal polyposis, multiple osteomas, skin fibroids, epidermoid cysts, and the presence of permanent and supernumerary dental elements [53,54,55].

## 4. Imaging

On a CT scan, an osteoma appears as a very radiodense lesion, similar in appearance to normal bone cortex, and mature osteomas may also demonstrate central marrow [56]. Osteomas are usually round or oval, with well-defined and smooth margins, without a perilesional halo [57]. CT is able to better define the epicenter of a bone lesion (medullary, cortical, periosteal, or parosteal) and its behavior in relation to adjacent structures, as a benign or aggressive growth pattern [58]. Specifically, osteomas can eventually determine bone expansion, a peculiarity that can help the differential diagnosis with idiopathic osteosclerosis [59]. In the literature, different CT findings are described according to the osteoma subtypes, as the ivory type is characterized by very dense bone with some small defined lucent areas, while the mature type shows an uneven bone density mixed with less dense areas, with an appearance similar to fibrous matrix [59,60]. CT is superior to conventional radiography, offering more details about the relationship between the osteoma and the adjacent structures [60]. Moreover, CT studies with 2D and 3D reconstructions provide great support for surgical planning, especially in cases of complex anatomical locations.

The last two decades have seen an increasing importance placed on cone beam computed tomography (CBCT) in diagnosis and treatment planning for maxillofacial district diseases. Smaller physical dimensions, lower costs, and lower radiation doses, when compared to traditional multi-detector CT scans (MDCT), have led to rapid expansion of CBCT scans [61,62,63,64,65,66,67]. Recently, technological advancements introduced the concept of dynamic automatic exposure control, in which exposure is adjusted during the acquisition of the image to optimize the radiation dose according to the size and mass of the patient. The doses of ionizing radiation administered by CBCT are generally 5–20 times lower, with the same volume irradiated, when compared to MDCT [68]. In particular, Ludlow et al. [62] compared the effective radiation dose of CBCT with a 64-slice MDCT for oral and maxillofacial imaging, concluding that with a medium field of view (FOV), the CBCT dose ranged from 69 to 560 μSv, whereas MDCT produced 860 μSv, indicating that the effective dose from a standard dental protocol scan with MDCT was 1.5–12.3 times greater than from a CBCT scan.

**Figure 8:**
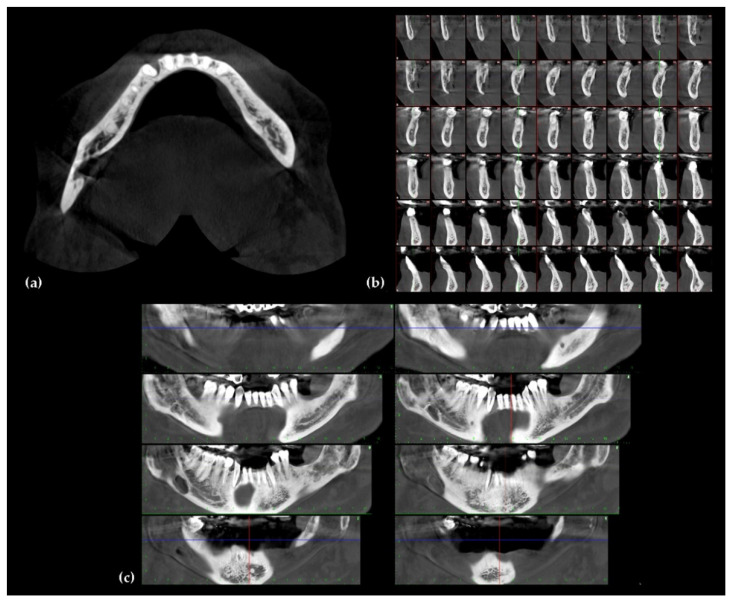
A cone beam computed tomography (CBCT) multiplanar reconstruction of lower jaw: (**a**) axial view, (**b**) sagittal view, and (**c**) coronal view.

CBCT scans can cover a large area of the facial skeleton, overcoming the limits of conventional radiography. Moreover, CBCT can be reformatted and viewed in multiplanar views (multiplanar reconstruction, or MPR) [66] (Figure 8). CBCT images may be comparable to MDCT images in terms of definition. Currently, neither MDCT nor CBCT can replace magnetic resonance imaging (MRI) for soft tissue evaluation [67]. Newer CBCT scans allow slice thickness to be as low as 0.1 mm, allowing better evaluation of ill-defined margins of bone tumors (e.g., osteomas) for presurgical evaluation or post-surgical follow-up [65]. CBCT images offer acceptably accurate measurements for osseous components, with less than 1% error when compared to the gold standard of unenhanced CTs of the skull [64,65,66].

To date, the literature on radiology of the oro-maxillofacial region is mainly represented by case series and transverse or prevalence studies, which do not provide substantial evidence for clinical decision-making; however, many literature reviews agree that CBCT should be considered as the method of choice for diagnosis of the dento-maxillofacial region [68,69,70,71,72].

A study by Hofmann et al. [73] compared five cone-beam CT (CBCT) scanners and three multi-slice low-dose CT (MSCT) scanners, in evaluating image quality and organ doses. Results proved that image quality was similar among the various systems tested, but they demonstrated distinct differences in organ dose levels. Interestingly, the lowest dose (0.03 mSv) was measured with a CBCT unit and the highest dose (8.30 mSv) with a different CBCT unit, proving that (depending on the model and setting use) MSCT radiation levels may be even lower than CBCT scan radiation levels. MDCT with optimized low-dose protocols may be considered as an alternative to CBCT in dento-maxillofacial evaluation, as it guarantees comparable image quality with considerable dose reduction, while also preserving soft-tissue detail [74,75].

The use of MRI in the evaluation of craniofacial osteomas is limited, due to the nature of the lesions. As an osteoma is a dense bone lesion, its evaluation is faster and more effective via CT [57]. Nevertheless, MRI can be used as a supplement to CT in the assessment of adjacent soft tissues and complications associated with an osteoma, such as inflammatory changes in mucosa in the case of an osteoma arising in paranasal sinuses [21,22,25,28,31,35,37].

Integrated 99m Tc-methylene diphosphonate single-photon emission computed tomography (SPECT/CT) is a nuclear medicine study that supplies both functional and anatomical information about the bone, playing a pivotal role as an osteoblastic biomarker for primary bone neoplasms such as osteomas [69]. An osteoma quantitative bone SPECT/CT shows a region of focal radiotracer uptake at the level of the radiodense lesion, providing an accurate functional evaluation of the lesion and supplying anatomical information that can be valuable for diagnosis [76,77,78,79,80,81]. The Tc-99m bone scintigraphy technique with SPECT/CT can be used as a diagnostic aid in cases of multiple osteomas (as in Gardner syndrome), as it is able to easily identify multiple foci of radiotracer uptake, facilitating detection and simplifying diagnosis [76,77,78,79,80,81,82]. Moreover, quantitative bone SPECT/CT can be useful in assessing the biologic growth activity of osteomas, establishing whether a lesion is still actively growing or is relatively inert, which can be helpful in determining the most appropriate management [83,84].

The differential diagnosis of osteomas includes several inflammatory and tumoral pathologies: exostosis, cemento-osseous dysplasia, Paget’s disease, chronic focal sclerosing osteomyelitis, osteoblastoma, ossifying fibroma, chondroma, osteosarcoma, fibrous dysplasia, and odontoma [7,10,85]. The differential diagnoses for osteomas and osteoblastomas can be challenging, as they are closely related pathologies. In this situation, the anatomical onset sites may be helpful, because osteoblastoma is more common in the jaw and occurs predominantly on the left side of the posterior mandible [85]. Osteoblastomas are usually larger in size and exhibit a more rapid rate of growth than osteomas. Radiographically, osteomas appear as radiopaque lesions with a reactive sclerosis of bone and a possible periosteal reaction, while osteoblastomas are radiolucent lesions [57,85].

## 5. Surgical Treatment

**Figure 9:**
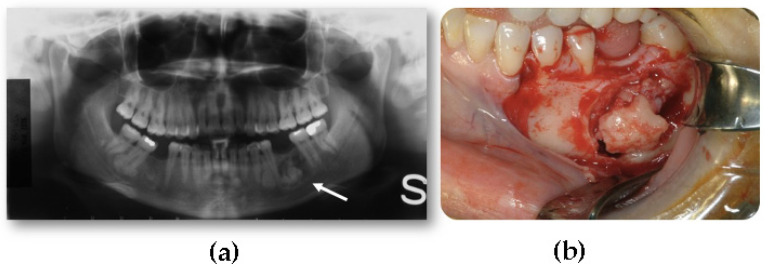
A case of a mandibular osteoma. (**a**) The panoramic radiograph demonstrates a focal radiopaque lesion (white arrow); (**b**) Osteoma’s surgical removal via the transoral approach at).

Even if there is still no unequivocal consensus, asymptomatic osteomas do not usually require surgery, but rather a “wait and see” strategy based on clinical and radiological follow-up, preferably with CBCT or low-dose MDCT [68,69,70,71,72,73,74,75]. Surgical treatment is only considered in cases of clinical worsening [86,87]. This approach is justified by the fact that osteomas have a slow growth pattern and rarely cause complications, as suggested by their benign nature [11]. Surgical resection is the gold standard treatment. It is based on a radical excision extending to the surrounding normal bone, with the contextual aim of achieving an optimal cosmetic result by choosing the most minimally invasive surgical treatment possible (Figure 9) [88,89,90].

In the case of mandibular osteomas, when only cosmetic changes are required therapy consists of simple lesion excision, while extraoral techniques are limited to bigger osteomas when more extensive exposure is required [91,92].

Larger lesions involving the maxilla may require extensive resective treatment followed by reconstruction with free flaps and/or cad-cam prostheses [86]. Lesions involving the paranasal sinuses can be treated via an endoscopic approach, with en-bloc excisions for lesions of small size and “piecemeal” resections for larger lesions.

In the last decade, computer-assisted surgery for jaw lesions has been demonstrated to achieve better outcomes when compared to traditional techniques [92,93,94]. This clinical improvement is due to the possibility of preoperative simulation of demolitive and reconstructive surgery. Image-based planning of surgical resection, combined with intraoperative navigation, has exhibited a great potential in the sphere of bone surgery and, in particular, has acquired a pivotal role in oncological cranio-maxillofacial surgery. In fact, pre-operative resection planning can be reproduced intraoperatively using surgical navigation systems that can be extremely precise during surgery, with the aim of better detecting fundamental anatomical structures (e.g., nerves, vessels, and muscles) to enable less demolitive surgical treatment.

**Figure 10:**
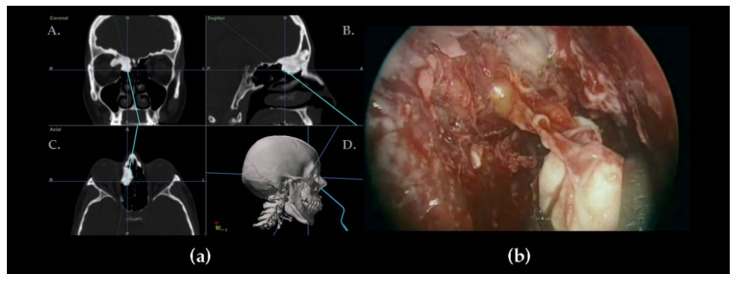
Navigation-guided endoscopic approach for ethmoid–orbital osteoma. (**a**) Navigation system screenshot showing an osteoma’s identification with a navigation pointer: A. coronal view, B. sagittal view, C. axial view, D. 3D reconstruction. (**b**) Endoscopic view during the osteoma’s removal via the trans-nasal approach.

In addition, surgical navigation, especially if associated with endoscopy, allows a surgeon to avoid open accesses, thus reducing surgical morbidity. This application is particularly efficient in osteomas in the ethmoid–orbital region (Figure 10).

Reconstructive surgery for an osteoma is quite infrequent, and is reserved for patients affected by large central osteomas with big mandibular or maxillary lesions, where reconstructive surgery could be mandatory. Recent medical literature shows that computer-assisted design and manufacturing techniques for jaw reconstruction are the best ways of obtaining better aesthetic and functional results [95,96].

## 6. Conclusions

An osteoma is a benign bone craniofacial lesion with a slow growth rate, mainly affecting the mandible. It is usually asymptomatic and detected as an incidental finding in imaging examinations performed for other reasons.

The gold standard imaging modality for investigating osteomas is CT, which can easily identify their “ivory like” appearance, enabling differentiation from other bone diseases in support of pre-surgical analysis. In this regard, the last two decades have seen a significant diffusion of CBCT in diagnosis and treatment planning of craniofacial osteomas, as it requires a lower radiation dose when compared with conventional MDCT, guarantees optimal spatial definition, and, at the same time, provides multi-planar reconstruction modality for an adequate pre- and post-treatment evaluation.

Treatment is usually performed during an advanced stage of the disease, particularly when an osteoma causes symptoms or functional and aesthetic issues.

## Data Availability

The data presented in this study are available on request from A.T. and F.R.
